# Bioequivalence of perampanel fine granules and tablets in healthy Japanese subjects 

**DOI:** 10.5414/CP203781

**Published:** 2020-09-01

**Authors:** Sari Shiba, Hisakuni Sekino, Kaeko Ishiba, Sanae Yasuda, Syuhei Inoue, Ken Kotaka, Larisa Reyderman, Naoki Uchida

**Affiliations:** 1 *Eisai Co., Ltd., Tokyo, Japan,*; 2 *Medical Corporation HOUEIKAI, Sekino Clinical Pharmacology Clinic, Tokyo, Japan, *; 3 *Eisai Inc., Woodcliff Lake, NJ, USA, *; 4 *School of Medicine, Showa University, Tokyo, Japan*

**Keywords:** bioequivalence, fine granules, formulation, perampanel, pharmacokinetics

## Abstract

Objective: Perampanel is an approved anti-seizure drug. A new formulation of perampanel fine granules (FG; 1% perampanel) has been developed for patients who are unable to take tablets. Bioequivalence between the 4-mg FG and tablet perampanel formulations, as well as their safety and tolerability, were assessed. Materials and methods: In this phase I, single-center, open-label, 2-period, 2-sequence, crossover, bioequivalence study (NCT03399734), healthy Japanese subjects were randomized to receive single doses of the 4-mg FG perampanel and 4-mg perampanel tablet (separated by a ≥ 6-week washout period). Plasma samples for perampanel concentration analysis were collected pre-dose and at intervals up to 168 hours post-dose. The maximum observed concentration (C_max_) and area under the concentration–time curve from time zero to 168 hours (AUC_(0–168h)_) were used to assess the bioequivalence of the two formulations. Results: The 90% confidence intervals (CIs) for the geometric mean ratio of test/reference for C_max_ and AUC_(0–168h)_ were within the bioequivalence criteria of 80 – 125% (C_max _90% CI 90.8%, 110%; AUC_(0–168h)_ 90% CI 98.2%, 112%; N = 21). 10/24 (41.7%) subjects with FG experienced ≥ 1 treatment-emergent adverse event (TEAE). The events were mild in severity and resolved within 4 hours of onset. There were no deaths, severe TEAEs, serious AEs, or TEAEs leading to study-drug withdrawal. Conclusion: Bioequivalence of 4-mg FG and 4-mg tablet of perampanel was demonstrated. Both perampanel formulations were generally safe and well tolerated. These data suggest that perampanel FG may be a suitable alternative formulation for patients with epilepsy who have difficulties taking perampanel tablets.

****What is known about this subject****

- A tablet formulation of perampanel (2 mg and 4 mg) is available in Japan as an adjunctive treatment and monotherapy for focal seizures (with or without focal to bilateral tonic-clonic seizures) and adjunctive treatment for generalized tonic-clonic seizures.
- A fine-granule formulation of perampanel in Japan would make perampanel accessible to patients who are not able to take tablets.
- Bioequivalence of a fine-granule formulation of perampanel to the tablet formulation must be demonstrated before the fine-granule formulation can be made available in Japan and other countries.

****What this study adds****

- Bioequivalence between a 4-mg fine-granule formulation and a 4-mg tablet formulation of perampanel was demonstrated following single dosing under fasted conditions in healthy Japanese adults. The single 4-mg dose of perampanel fine granules and tablet was generally safe and well tolerated.
- The results from this study suggest that the use of a fine-granule formulation of perampanel may be a suitable alternative for patients who have difficulty taking perampanel tablets.

## Introduction 

Perampanel, a first-in-class, selective, non-competitive α-amino-3-hydroxy-5-methyl-4 isoxazolepropionic acid (AMPA) receptor antagonist [1], is a once-daily, oral, anti-seizure drug for focal seizures (previously referred to as partial-onset seizures) with or without focal to bilateral tonic-clonic seizures (previously referred to as secondarily generalized seizures), and for generalized tonic-clonic seizures (GTC; previously referred to as primary generalized tonic-clonic seizures) [2, 3, 4]. The safety and efficacy of perampanel in adolescent and adult patients with focal seizures or GTC seizures have been well documented in several double-blind, randomized, placebo-controlled phase III studies [5, 6, 7, 8, 9], and long-term tolerability and improvements in seizure outcomes were demonstrated in their associated phase III extension studies [10, 11]. 

Following oral administration, perampanel is rapidly absorbed (time from dosing to peak plasma concentration of 0.5 – 2.5 hours under fasted conditions), and plasma concentrations of perampanel increase in direct proportion to administered doses over the clinically relevant dose range (2 – 12 mg) [2, 3]. Although the rate of perampanel absorption is slowed by food co-ingestion, the extent absorbed remains unchanged; therefore, perampanel can be administered without regard to meal times [2, 3]. Perampanel is primarily metabolized via cytochrome P450 3A, with an elimination half-life of ~ 105 hours in healthy subjects [2, 3]. 

Perampanel is approved and available under the tradename Fycompa as oral tablets (2, 4, 6, 8, 10, and 12 mg) and oral suspension (0.5 mg/mL) in the US and EU [2, 3]. The tablet formulation of perampanel was introduced first [12], followed by perampanel oral suspension (based on completion of a study where bioequivalence of the oral suspension to the tablet formulation was demonstrated under fasted conditions) [2], because non-tablet oral formulations of the drug were required to meet the needs of patients who are unable to or prefer not to swallow a solid oral dosage form, especially pediatric and elderly patient populations. In Japan, perampanel 2-mg and 4-mg tablets are available [13]. A fine-granule formulation has been developed as a non-tablet oral form, containing 1% perampanel by mass, and was approved in Japan in January 2020 [14]; 0.4 g of perampanel fine granules contains 4-mg perampanel, hereafter referred to as “4-mg fine granules”. 

We present here the results from a phase I study that aimed to demonstrate bioequivalence between the 4-mg fine-granule and tablet formulations of perampanel under fasted conditions in healthy Japanese subjects. 

## Materials and methods 

### Study overview

This was a phase I, single-center, open-label, 2-period, 2-sequence, crossover, bioequivalence study in healthy Japanese subjects (protocol: E2007-J081-053; ClinicalTrials.gov identifier: NCT03399734) and was carried out between December 18, 2017, and March 9, 2018. The study was approved by the Houeikai Institutional Review Board, Tokyo, Japan, and was carried out in accordance with Japan’s Good Clinical Practice guidelines and the World Medical Association Declaration of Helsinki. Written informed consent was obtained from all subjects prior to screening. 

The primary objective of the study was to demonstrate bioequivalence between a single 4-mg dose of fine granules of perampanel and a single 4-mg tablet of perampanel. The secondary objective was to evaluate and compare the safety and tolerability of a 4-mg dose of fine granules of perampanel with a 4-mg tablet of perampanel. 

### Subjects

The study was carried out in healthy, non-smoking Japanese subjects aged ≥ 20 and ≤ 45 years with a body mass index of ≥ 18.5 and < 25.0 kg/m^2^. Smokers had to discontinue smoking from the screening visit to before the first dosing. Key exclusion criteria included: clinically significant illness that required medical treatment within 8 weeks, or a clinically significant infection that required medical treatment within 4 weeks before the first dosing; history of gastrointestinal surgery that had potential to affect pharmacokinetic (PK) profiles of perampanel; and, subjects who were HIV positive and/or had active viral hepatitis B or C, or syphilis at screening. Females who were breast feeding or pregnant at screening or baseline were not eligible for the study. Females of child-bearing potential were required to use a highly effective form of contraception for 28 days before study entry, throughout the entire study period, and for 28 days after the final dose of study drug. 

### Study design and procedures

The study consisted of a pre-randomization phase and a randomization phase. The pre-randomization phase consisted of a screening period (between day –28 and day –2) and baseline period 1 (day –1, i.e., the day before study-drug administration in treatment period 1). Subjects who completed baseline period 1 and who met the inclusion/exclusion criteria could enter the randomization phase. The randomization phase consisted of treatment period 1, baseline period 2, treatment period 2, and a follow-up period. Treatment periods 1 and 2 were separated by a washout period of at least 6 weeks. The follow-up period took place on day 15, which was 2 weeks after the last dose of study drug on day 1 of treatment period 2. 

Subjects were randomized (1 : 1) on day 1 of treatment period 1 to one of two treatment schedules: a single 4-mg tablet on day 1 of treatment period 1, followed by 4-mg fine granules on day 1 of treatment period 2, or 4-mg fine granules on day 1 of treatment period 1, followed by a 4-mg tablet on day 1 of treatment period 2. The dose selection of 4-mg perampanel was made in line with Japanese guidelines on bioequivalence studies [15] and on the basis of the 4-mg tablet being the highest-available perampanel tablet strength in Japan. Perampanel was administered with 200 mL water following an overnight fast (≥ 10 hours); subjects continued to fast for 4 hours post-dose. Water was permitted except for 1 hour before and after perampanel administration. 

### Pharmacokinetic assessments

Blood samples for PK analyses were collected pre-dose and at 0.25, 0.5, 0.75, 1, 1.25, 1.5, 2, 3, 4, 6, 8, 12 hours (day 1), 24, 36 hours (day 2), 48 hours (day 3), 72 hours (day 4), 120 hours (day 6), and 168 hours (day 8) post-dose in treatment periods 1 and 2, in line with Japanese guidelines on bioequivalence studies of generic products [15]. Plasma concentrations of perampanel were measured using a validated liquid chromatography with tandem mass spectrometry assay [16]. 

PK parameters were derived by noncompartmental analysis of perampanel plasma concentration-time data. Primary PK parameters were the maximum observed concentration (C_max_) and area under the concentration-time curve from time zero to 168 hours (AUC_(0–168h)_). Secondary PK parameters included time at which the highest drug concentration occurs (t_max_), area under the concentration-time curve from time zero extrapolated to infinity (AUC_(0–inf)_), and the terminal elimination phase half-life (T_1/2_). The PK analysis set comprised subjects who received study drug, completed both treatment periods 1 and 2, and had sufficient plasma perampanel concentration data to be evaluated for bioequivalence in both periods. 

### Bioequivalence evaluations

The primary PK parameters (C_max_ and AUC_(0–168h)_) of perampanel were compared between 4-mg fine-granule and tablet formulations, using a mixed linear model of logarithmically transformed values with fixed effects for treatment, period, and sequence, and a random effect of subject. Two-sided 90% confidence intervals (CIs) for the geometric mean ratio of test/reference for C_max_ and AUC_(0–168h)_ were estimated. If each of the two-sided 90% CIs fell within 80 – 125%, it was to be concluded that the fine-granule formulation was bioequivalent to the tablet formulation. Similar statistical analyses were conducted for AUC_(0–inf)_. Values for t_max_ and the T_1/2_ were summarized for each formulation. In addition, point estimates and two-sided 90% CIs for differences in median t_max_ values were calculated using the Hodges-Lehmann estimator method. A statistically significant difference in t_max_ was to be concluded if the two-sided 90% CIs did not contain zero. 

### Safety assessments

Safety was assessed via monitoring and recording all adverse events (AEs) and serious AEs (SAEs); laboratory evaluations for hematology, chemistry, and urine values; periodic measurement of vital signs, body weight, and electrocardiograms (ECGs); and physical examinations. Period 1 treatment-emergent AEs (TEAEs) were defined as any AE that occurred at any time during the period from the study treatment in treatment period 1 to 28 days after study treatment. Period 2 TEAEs were any AE that occurred at any time during the period from the study treatment in treatment period 2 to 28 days after study treatment. TEAEs were summarized by formulation. The safety analysis set comprised subjects who received ≥ 1 dose of study drug. 

## Results 

### Subjects 

A total of 24 male subjects were randomized and included in the overall safety analysis set. All 24 subjects completed treatment period 1, and 21 subjects completed treatment period 2. Three subjects who received 4-mg fine granules in treatment period 1 discontinued prior to treatment with the 4-mg tablet in treatment period 2. In treatment period 2, 12 subjects who received the 4-mg fine granules and 9 subjects who received the 4-mg tablet completed this period. Details of subject disposition and demographics are shown in Table 1 and Table 2, respectively. 

### Pharmacokinetic analysis

The PK analysis set comprised 21 subjects who completed both treatments and had sufficient plasma perampanel concentrations for PK evaluation. Three subjects had quantifiable pre-dose plasma concentrations of perampanel in treatment period 2; however, data from these subjects were included in the PK and statistical analysis because these concentrations were ≤ 5% of their respective C_max_, in line with the US Food and Drug Administration guideline on bioequivalence [17]. 

**Figure 1.**
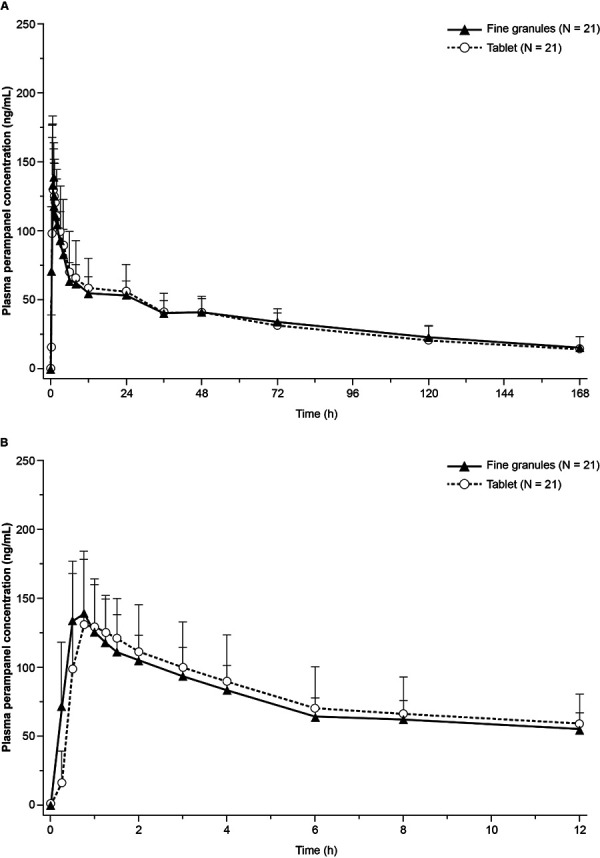
Perampanel mean (+ SD) plasma concentration-time profiles up to (A) 168 hours, and (B) 12 hours after single-dose administrations of 4-mg fine granules and 4-mg tablet on a linear scale (PK analysis set). PK = pharmacokinetic; SD = standard deviation.

Mean plasma concentration-time profiles of perampanel up to 168 hours and 12 hours after a single dose of the 4-mg fine granules and the 4-mg tablet are presented in Figure 1. The PK profiles of both fine granules and tablet declined in a biphasic manner with a mean T_1/2_ of 87.9 hours (fine granules) and 89.5 hours (tablet). The PK profile for the 4-mg fine granules was superimposable with that for the 4-mg tablet. Perampanel PK parameters are summarized in Table 3. 

### Bioequivalence analysis

Results of the bioequivalence statistical analysis are shown in Table 4. For C_max_, the geometric mean ratio of the 4-mg fine granules to the 4-mg tablet was 100%, and the 90% CI was 90.8 – 110%. For AUC_(0–168h)_, the geometric mean ratio of the 4-mg fine granules to the 4-mg tablet was 105%, and the 90% CI was 98.2 – 112%. The 90% CIs for both C_max_ and AUC_(0–168h)_ fell within the bioequivalence criteria of 80 – 125%, and therefore the bioequivalence criteria were satisfied for both parameters. 

### Safety analysis

The overall safety analysis set comprised 24 subjects. 24 subjects were included in the safety analysis set for the 4-mg fine-granule formulation, and 21 subjects were included in the safety analysis set for the 4-mg tablet formulation. 

Overall, 10 (41.7%) subjects in the 4-mg fine-granule group experienced at least 1 TEAE, compared with 3 (14.3%) subjects in the 4-mg tablet group. TEAEs were dizziness (7 (29.2%) subjects and 2 (9.5%) subjects in the 4-mg fine-granule and tablet group, respectively) and somnolence (6 (25.0%) subjects and 2 (9.5%) subjects in the 4-mg fine-granules and tablet groups, respectively). All TEAEs in both groups were considered related to the study drug but were reported as mild in severity. All TEAEs had an onset time of within 1 hour of drug administration and resolved within 4 hours of onset without treatment. 

There were no deaths, severe TEAEs, or SAEs, and no subjects were withdrawn from the study due to TEAEs. Three subjects withdrew prior to treatment in treatment period 2 due to increased lactate dehydrogenase, aspartate aminotransferase, and alanine aminotransferase (n = 1); pyrexia (n = 1); and increased white blood cell count (n = 1). However, these events leading to withdrawal were not classified as AEs due to their time of onset (41 – 42 days post-dose) occurring after the protocol-defined AE collection period. All these events returned to normal levels by follow-up visits. 

There were no clinically significant laboratory values that were judged to be an AE by the investigator, and no changes of clinical concern in vital signs, body weight, or ECGs. 

## Discussion 

In the US and EU, perampanel is approved in both tablet and oral suspension formulations for the treatment of focal seizures (with or without focal to bilateral tonic-clonic seizures) and GTC seizures [2, 3]. In Japan, granules and oral dry-syrup formulations are often used as an alternative to tablets. For example, the anti-seizure drugs levetiracetam and lacosamide are available in tablet, intravenous (IV), and liquid oral solution/syrup formulations in the US and EU [18, 19, 20, 21, 22], while in Japan, these anti-seizure drugs are available in tablet, IV, and dry-syrup formulations only [23, 24]. A fine-granule formulation of perampanel has been developed for use in Japan and other countries, and has recently been approved in Japan. The incidence of epilepsy is higher in children and the elderly compared with adults [25, 26]. Therefore, the fine-granule formulation is likely to be of particular benefit for pediatric and elderly patients who may require non-tablet oral formulations. 

In line with regulatory guidance in Japan, a bioequivalence study of the currently available tablet formulation and the fine-granule formulation of perampanel under fasted conditions was required to investigate the bioequivalence of the two formulations. As such, the current study aimed to evaluate the bioequivalence of the 4-mg fine-granule formulation of perampanel and the approved 4-mg tablet formulation of perampanel. Both C_max_ and AUC_(0–168h)_ satisfied the bioequivalence criteria, indicating bioequivalence of the 4-mg fine-granule and 4-mg tablet formulation of perampanel. This finding is in line with a previous bioequivalence study of perampanel, which demonstrated bioequivalence of single doses of 12-mg perampanel tablet formulation with 12-mg perampanel oral suspension [27]. 

The single dose of the 4-mg fine granules, as well as the single dose of the 4-mg tablet, was generally safe and well tolerated in healthy Japanese subjects, and no new safety concerns were identified. In this study, the incidences of dizziness and somnolence in the 4-mg fine-granule group (29.2% and 25.0%, respectively) were higher compared with the 4-mg tablet group (9.5% and 9.5%, respectively), although the reason for this apparent difference is unknown. However, all these events reported in both groups were mild in severity, had an onset within 1 hour of drug administration, and resolved within 4 hours of onset. Furthermore, dizziness and somnolence are the most frequently reported AEs associated with perampanel, although the incidence of these AEs in the current study was lower compared with a previous single-dose clinical study of perampanel tablets in healthy Japanese subjects (study E2007-J081-010; Eisai data on file). In study E2007-J081-010, the incidences of dizziness and somnolence were 33.3% (2/6 subjects in the 2-mg tablet group) and 50.0% (3/6 subjects in the 4-mg tablet group), respectively. The data from the current study are also consistent with the known safety profile of the tablet formulation of once-daily perampanel in focal seizures (with or without focal to bilateral tonic-clonic seizures) or generalized tonic-clonic seizures [5, 6, 7, 8]. In addition, there were no deaths, severe TEAEs, SAEs, or TEAEs leading to study-drug withdrawal during the study. Furthermore, no clinically important changes that were judged to be an AE by the investigator in clinical laboratory parameters, vital signs, body weight, or ECG findings were observed in this study. 

## Conclusion 

Bioequivalence of 4-mg fine granules of perampanel with the 4-mg tablet of perampanel was concluded in this study (via C_max_ and AUC_(0–168h)_ parameters). The single 4-mg dose of fine granules of perampanel was generally safe and well tolerated, and no new safety concerns were identified in healthy Japanese subjects. Overall, these data suggest that fine granules of perampanel may be a suitable alternative formulation for patients who have difficulties taking perampanel tablets. 

## Acknowledgment 

Medical writing support, under the direction of the authors, was provided by Kirsty Muirhead, PhD, and Lisa Moore, PhD, of CMC AFFINITY, McCann Health Medical Communications, funded by Eisai Inc., in accordance with Good Publication Practice (GPP3) guidelines. 

## Funding 

This study was funded by Eisai Co., Ltd. 

## Conflict of interest 

SS, SI, KI, KK, and SY are employees of Eisai Co., Ltd., Japan. LR is an employee of Eisai Inc., USA. HS and NU have no relevant disclosures. 

**Table 1.** Subject disposition (all randomized subjects).

	4-mg fine granules (N = 24)	4-mg tablet (N = 24)
Treated, n (%)	24 (100)	21 (87.5)
Completed the study, n (%)	24 (100)	21 (87.5)
Discontinued from the study, n (%)	0	3 (12.5)
Discontinued before study treatment in treatment period 2, n	0	3
Primary reason for discontinuation, n (%)		
Other^a^	0	3 (12.5)

^a^Reasons for discontinuations from study were: increased lactate dehydrogenase, aspartate aminotransferase, and alanine aminotransferase (n = 1); pyrexia (n = 1); and increased white blood cell count (n = 1).

**Table 2.** Subject demographics (overall safety analysis set and PK analysis set).

	Overall safety analysis set (N = 24)	PK analysis set (N = 21)
Age (years)^a^		
Mean (SD)	33.3 (8.19)	33.1 (8.20)
Median (min, max)	33.5 (21, 45)	33.0 (21, 45)
Sex, n (%)		
Male	24 (100)	21 (100)
BMI (kg/m^2^)		
Mean (SD)	21.38 (1.336)	21.40 (1.329)
Median (min, max)	21.30 (18.5, 24.2)	21.30 (18.5, 24.2)

Percentage is based on the total number of subjects with non-missing values in the relevant analysis set. ^a^Age is calculated at date of informed consent. BMI = body mass index; max = maximum; min = minimum; PK = pharmacokinetic; SD = standard deviation.

**Table 3.** Summary of PK parameters of perampanel after single-dose administrations of 4-mg fine granules and 4-mg tablet (PK analysis set).

PK parameter	4-mg fine granules (N = 21)	4-mg tablet (N = 21)
C_max_ (ng/mL)		
Mean (SD)	152 (28.3)	154 (42.6)
Geometric mean (CV%)	149 (19.9)	149 (28.5)
AUC_(0–168h)_ (ng×h/mL)		
Mean (SD)	5,770 (1,340)	5,620 (1,600)
Geometric mean (CV%)	5,630 (22.9)	5,420 (27.8)
AUC_(0–inf)_ (ng×h/mL)		
Mean (SD)	8,040 (2,750)	8,000 (3,770)
Geometric mean (CV%)	7,610 (35.5)	7,320 (43.6)
t_max_ (h), median (min, max)	0.75 (0.50, 3.00)	0.75 (0.50, 2.00)
T_1/2_ (h)		
Mean (SD)	87.9 (32.4)	89.5 (49.4)
Geometric mean (CV%)	81.8 (41.2)	78.2 (56.7)

AUC_(0–168h)_ = area under the concentration–time curve from time zero to 168 hours; AUC_(0–inf)_ = area under the concentration–time curve from time zero extrapolated to infinity; C_max_ = maximum observed concentration; CV% = percent coefficient of variation; h = hours; PK = pharmacokinetic; max = maximum; min = minimum; SD = standard deviation; t_max_ = time at which the highest drug concentration occurs; T_1/2_ = terminal elimination phase half-life.

**Table 4.** Statistical analysis of PK parameters of perampanel after single-dose administrations of 4-mg fine granules and 4-mg tablet (PK analysis set).

PK parameter	Geometric means		Geometric mean ratio (%) (test : reference)	90% CIs (%) (lower, upper)
	4-mg fine granules (test) (N = 21)	4-mg tablet (reference) (N = 21)		
C_max_ (ng/mL)	149	149	100	90.8, 110
AUC_(0–168h)_ (ng×h/mL)	5,630	5,420	105	98.2, 112

AUC_(0–168h)_ = area under the concentration–time curve from time zero to 168 hours; CI = confidence interval; C_max_ = maximum observed concentration; PK = pharmacokinetic.
